# Characterizing CD133 and NANOG Expression in Melanoma: Associations with Histological and Epidemiological Parameters

**DOI:** 10.3390/medicina60101658

**Published:** 2024-10-10

**Authors:** Adrian-Horațiu Sabău, Raluca Niculescu, Iuliu-Gabriel Cocuz, Andreea-Cătălina Tinca, Andreea Raluca Szöke, Bianca Andreea Lazar, Diana Maria Chiorean, Corina Eugenia Budin, Alexandru Nicușor Tomuț, Ovidiu Simion Cotoi

**Affiliations:** 1 Doctoral School of Medicine and Pharmacy, University of Medicine, Pharmacy, Sciences and Technology “George Emil Palade” of Targu Mures, 540142 Targu Mures, Romania; adrian-horatiu.sabau@umfst.ro; 2 Pathology Department, Mures Clinical County Hospital, 540011 Targu Mures, Romania; iuliu.cocuz@umfst.ro (I.-G.C.); andreea-catalina.tinca@umfst.ro (A.-C.T.); andreea-raluca.szoke@umfst.ro (A.R.S.); ohii.bianca@yahoo.com (B.A.L.); diana.chiorean@umfst.ro (D.M.C.); ovidiu.cotoi@umfst.ro (O.S.C.); 3 Pathophysiology Department, University of Medicine, Pharmacy, Sciences and Technology “George Emil Palade” of Targu Mures, 540142 Targu Mures, Romania; corina.budin@umfst.ro; 4 Pneumology Department, Clinical County Hospital Mureș, 540136 Targu Mures, Romania; 5 Faculty of Medicine, University of Medicine, Pharmacy, Sciences and Technology “George Emil Palade” of Targu Mures, 540142 Targu Mures, Romania; nicusortomut19@gmail.com

**Keywords:** melanoma, CD133, NANOG, immunohistochemistry

## Abstract

*Background/Objectives*: Melanoma is an aggressive skin malignancy, and the majority of deaths associated with melanoma result from malignant skin lesions. Our study aims to evaluate the expression of the markers CD133 and NANOG, associated with tumor stem cells, and to analyze their link with epidemiological and histological parameters, thus contributing to early diagnosis and the development of targeted therapies. *Methods*: We performed a retrospective study in the Mureș Clinical County Hospital, Romania, which included 66 cases of melanoma: 50 primary cutaneous melanomas, 10 metastases, and 6 local recurrences. CD133 and NANOG marker expression was assessed by immunohistochemistry and quantified using the H score. Statistical analyses were applied to determine the correlations between marker expression and clinicopathological parameters. *Results*: CD133 expression was identified in six cases (12%) of primary melanoma, with a mean H-Score of 29, and was associated with an increased Breslow index and a higher number of mitoses. NANOG expression was positive in 30 cases (60%) of primary melanoma, with a median H-Score of 15 and with increased expression observed in cases with pagetoid migration and lesions in situ. In metastases, eight cases (80%) were positive for NANOG and four (40%) for CD133. Local recurrences showed positive expression for NANOG in four cases (66%). *Conclusions*: The expression of CD133 and NANOG markers highlights the role of tumor stem cells in melanoma progression. Early identification of these markers could improve diagnosis and treatment, including the application of targeted therapies.

## 1. Introduction

Melanoma is a malignant skin tumor that arises from melanocytic cells, which are the cells responsible for melanin production, located in the basal layer of the epidermis. Despite accounting for only 1% of all skin malignancies, melanoma is responsible for the vast majority of deaths related to skin malignancies. This is due to tumor aggressiveness, frequent distant metastases, and frequent local recurrences. In recent decades, the incidence of melanoma has been increasing, especially among the Caucasian population and among the populations of Australia and New Zealand, due to prolonged exposure to ultraviolet (UV) radiation [1]. Prolonged exposure to UV radiation, both through direct exposure to the sun and through the use of tanning beds, plays a key role in the occurrence of genetic mutations that contribute to the appearance of tumor cells. The changes encountered are at the level of cellular DNA that induces structural alterations, but also triggers inflammatory processes and remodeling [2].

In terms of pathophysiology, melanoma presents a variety of morphological changes and biological aspects, through which it imitates or simulates other types of tumors. For this reason, the diagnosis of these tumors is always a real challenge. Immunohistochemistry plays a very important role in the diagnosis of melanoma and its differentiation from other melanocytic lesions [3]. The interpretation of specific markers such as S100, SOX-10, Melan-A, HMB-45, and PRAME can be challenging, making it essential to correlate histopathological data with clinical information and other histological and molecular findings [4,5].

Apart from conventional, morphological, and immunohistochemical diagnostic elements, recently discovered molecular biology techniques (BRAF, NRAS, and KIT gene mutations and the alteration of MAPK and PI3K-AKT cell signaling pathways) have opened a new chapter in melanoma diagnosis, and have contributed to the development of targeted therapies. These new techniques not only result in a more accurate diagnosis but have a direct impact on the prognosis and quality of life of patients [6,7,8].

A new area of research in the pathogenesis of melanoma is represented by tumor stem cells (CSCs). Their presence has been intensively studied at the primary tumor level, but also at the level of metastases and local recurrences. These cells are considered to be only a small fraction of the entire tumor population and are responsible for multiple tumor properties, such as initiation of the tumor process, tumor progression, the occurrence of metastases and recurrence of post-therapy or post-surgical excision, and resistance to therapy [9,10,11]. From the perspective of cutaneous melanoma, the most convenient method for identifying tumor stem cells is immunohistochemistry through specific markers [12].

One of the most studied immunohistochemical markers is CD133 (Prominin-1), a transmembrane glycoprotein present on the surface of cancer stem cells. The presence of this glycoprotein on tumor cells is associated with increased self-renewing capacity and treatment resistance [13]. Another marker recently studied is ALDH1, an enzyme involved in cellular metabolism, which provides enhanced protection against oxidative stress. Its expression has been associated with increased tumor aggressiveness, a more reserved prognosis, and reduced effectiveness of BRAF inhibitor therapies [14].

NANOG is another marker present in cancer stem cells. It is a cellular transcription factor responsible for self-renewal, apoptosis resistance, and cellular adaptation to the microenvironment, whether normal or tumor-related [10]. Due to its properties, NANOG is also a therapeutic target; inhibiting its signaling pathways has been shown to reduce tumor proliferation and provide a more efficient therapeutic response [15].

The primary involvement of the CD133 protein in cutaneous melanoma is represented by vasculogenic mimicry (VM), which constitutes a mechanism by which tumor cells show changes that lead to the appearance of phenotypes similar to endothelial cells and form vascular structures without the involvement of true endothelial cells. Unlike the angiogenesis process, this process is autonomous and characteristic of tumor stem cells. At the same time, CD133+ cells secrete vascular growth factors (VEGFs) that interact with receptors (VEGFR1) on the surface of cells involved in the vasculogenic mimicry (VM) process. Through this process, blood and nutrients are supplied to tumors, thus facilitating autonomous growth but also the possibility of vascular invasions [16]. NANOG, being a transcription factor highly involved in cell pluripotency, has been shown to play an essential role in achieving Epithelial–Mesenchymal Transition (EMT). In cases of melanoma, it has an important role in suppressing epithelial markers, e.g., E-cadherin, responsible for maintaining cell junctions. Thus, cellular mobility is increased, and the ability to invade the surrounding tissues is also increased. Moreover, by changing the phenotype through the BCL-2 way, the cells end up being resistant to apoptosis, thus avoiding the action of the immune system and conventional therapies [17]. NANOG can also stimulate autophagy, a process by which tumor cells can degrade and recycle certain components under stress conditions (in the case of therapy). Thus, tumor cells can protect themselves from the toxic effects of chemotherapy and can replace the elements degraded by the therapy [13]. Therefore, through these properties, cells positive for these two markers not only define this cell sub-population but also actively participate in the development, invasion and therapy resistance.

Thus, through our study, we have evaluated the immunohistochemical expression of CD133 and NANOG markers at the level of tumor cells in cutaneous melanoma and performed an analysis of the expression of the two markers and the epidemiological, histological, and biological parameters of the cases included in the study.

## 2. Materials and Methods

A retrospective observational study was conducted in the pathology department of the Mures Clinical County Hospital, Romania, which included 50 cases of primary cutaneous melanoma and 16 cases of melanoma metastases. All cases were diagnosed within the Clinical Pathology Department and surgical excision was performed in the departments of Surgery and Plastic and Reconstructive Surgery in our hospital between 2021 and 2023. The inclusion criteria encompassed cases with a complete histopathology report (containing all parameters followed in the study) and an immunohistochemistry profile (SOX-10, S100, HMB-45, MELAN-A, and Ki-67), as well as immunohistochemical reactions performed with anti-CD133 and anti-NANOG antibodies. The exclusion criteria comprised incomplete histopathology reports or missing immunohistochemical profiles.

The first step in the protocol involved preparing hematoxylin-eosin (HE) sections following standard procedures. On the HE-stained slides, histological parameters were measured, including histological subtype, Breslow index, presence or absence of ulceration, Clark invasion level, presence or absence of lymphovascular and perineural invasion, presence and type of intratumoral inflammatory infiltrate, mitotic count, presence or absence of in situ lesions, and pagetoid migration. In cases with large amounts of melanin pigment, depigmentation was performed to avoid misinterpretation. Immunohistochemistry (SOX-10, S100, HMB-45, and MELAN-A) was performed using an automated method (Benchmark GX; Ventana Medical Systems Inc., Tucson, AZ, USA) to confirm the diagnosis and distinguish melanoma from other melanocytic lesions.

The second step of the protocol involved manual immunohistochemical reactions for CD133 (Recombinant Anti-CD133 antibody EPR16508 ab222782) and NANOG (Recombinant Anti-Nanog antibody EPR2027(2) ab109250) markers. The technical steps included deparaffinization, rehydration of slides, rinsing with distilled water, antigen retrieval via microwave heating for 20 min in a pH = 9 solution, slow cooling for 20 min, rinsing with distilled water, blocking endogenous peroxidase for 10 min, rinsing with TBS, blocking nonspecific binding with Protein Block, applying the primary antibody (CD133 dilution 1:1000/NANOG dilution 1:100) overnight at 4 °C, washing with TBS, applying the secondary antibody (mouse anti-rabbit HRP; Agilent-Dako, Santa Clara, CA, United States), and developing with 33′-diaminobenzidine (DAB). The slides were counterstained with hematoxylin as per the manufacturer’s guidelines. External controls were performed on normal renal tissue for CD133 and on primary testicular seminoma for NANOG.

The third stage involved quantifying the immunohistochemical reactions for CD133 and NANOG using the H-Score, a widely used method in cancer immunohistochemistry, particularly in breast cancer. This semi-quantitative method accounts for both reaction intensity and the percentage of positive cells. The H-Score is calculated by multiplying the percentage of weakly positive cells by 1, moderately positive cells by 2, and strongly positive cells by 3, and then summing these values. The maximum score is 300, while the minimum score is 0 (indicating no reaction).

Statistical analysis was performed using MS Excel 2023 and IBM SPSS v.27.0. Grubbs’ test was used to identify and eliminate outliers, followed by the Kolmogorov–Smirnov test to determine data normality. For parametric data, Pearson’s correlation or the Student’s *t*-test was used, while for non-parametric data, the Wilcoxon or Mann–Whitney U tests were applied.

This study was conducted in accordance with the Declaration of Helsinki and was approved by the hospital’s ethics committee (protocol number 2200/15 March 2021).

## 3. Results

According to the inclusion and exclusion criteria, a total of 66 cases were included in the study. Fifty cases were represented by primary cutaneous melanomas, ten cases were distant metastases, and six cases were local recurrences in patients whose initial surgical excision was complete with safe surgical margins. Based on these three categories, we formulated and analyzed the results further.

**Figure 1:**
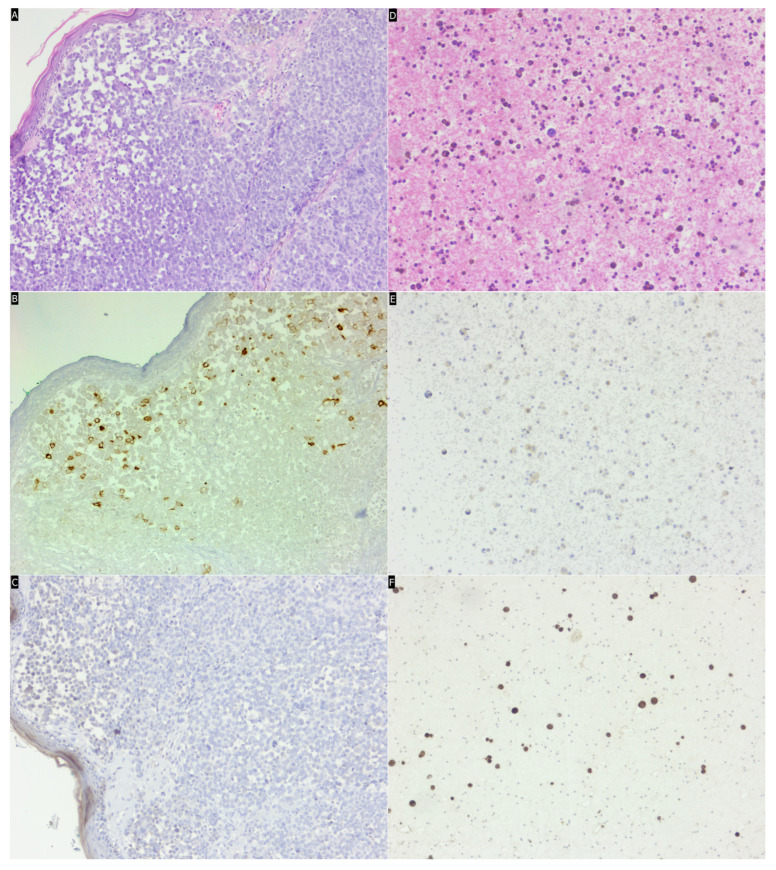
(**A**–**C**) A case of primary cutaneous nodular melanoma, stage pT4b (Breslow index greater than 4 mm and surface ulceration). (**A**) The HE stain (×20) reveals tumor proliferation with a nodular architecture, composed of epithelioid cells with eosinophilic cytoplasm and enlarged, pleomorphic nuclei. (**B**) Immunohistochemical reaction with anti-CD133 antibodies (×20); focally positive (H-Score 5 × 3 + 5 × 2 + 5 × 1 = 30). (**C**) Immunohistochemical reaction with anti-NANOG antibodies (×20); negative (H-Score 0). (**D**–**F**) A case of pleural melanoma metastasis. (**D**) Cytoblock in HE stain (×20) shows normal or reactive mesothelial cells, inflammatory cells, predominantly lymphocytes and plasma cells, along with isolated tumor cells with eosinophilic cytoplasm, irregular, pleomorphic nuclei, and prominent nucleolus. (**E**) Immunohistochemical reaction with anti-CD133 antibodies (×20); focally positive at the membrane level (H-Score 0 × 3 + 5 × 2 + 5 × 1 = 15). (**F**) Immunohistochemical reaction with anti-NANOG antibodies (×20); focally positive at the nuclear level (H-Score 30 × 3 + 10 × 2 + 5 × 1 = 115).

When analyzing the H-Score for the immunohistochemical reaction with anti-NANOG antibodies in the group of patients with primary cutaneous melanoma, 30 patients presented positive results (H-Score > 0). Among the 30 positive cases, an outlier (H-Score = 120) was identified based on Grubbs’ test. The minimum score was 5, the maximum score was 85, and the median score was 15, which was used as the cutoff between patients with High H-Score and Low H-Score. A slight increase in the average age at diagnosis, average Breslow index, and presence of pagetoid migration and in situ lesions was observed in these patients. All data were tested for normality, and specific tests were applied; no statistically significant correlation was identified between the analyzed data and the H-Score for any of the three categories analyzed (Table 1 and Figure 1).

One particular observation in this study was the presence of eight cases of superficial melanoma, all of which fell into the High H-Score category for the NANOG marker (Table 1).

An analysis of the H-Score for the immunohistochemical reaction with anti-NANOG antibodies in the group of patients with melanoma metastases (n = 10) determined that eight cases were positive and two cases were negative. The maximum score was 125, the minimum score was 5, and the average score was 43. Of these eight positive cases, five were metastases to lymph nodes (three in the axillary region and two in the inguinal region), with the primary lesion located on the extremities. The remaining three positive cases were pleural, hepatic, and intestinal metastases, all with the primary lesion located on the back. Six of the positive cases were pT4b at the initial diagnosis, and the remaining two were pT3b, with all six cases being nodular melanomas.

Regarding the expression in the group of patients with local recurrences (n = 6), four cases were positive and two were negative. The maximum score was 60, the minimum score was 5, and the average score was 31. All four positive cases were pT4b at the initial diagnosis, and all were nodular melanomas (Table 2).

Regarding the immunohistochemical expression for anti-CD133 antibodies in the group of patients with primary cutaneous melanoma, six positive cases were identified. The minimum score was 20, the maximum score was 40, and the average score was 29. The Grubbs’ test did not identify any outliers, and the Student’s *t*-test revealed statistically significant differences in the average Breslow index (*p*: 0.022035) and the average number of mitoses per 10/HPF (*p*: 0.001868). Additionally, a slight increase in the Ki-67 proliferation index was observed, although no statistically significant difference was found (*p*: 0.0320624). All six cases were nodular melanomas (Table 3 and Figure 1).

In the group of patients with melanoma metastases (n = 10), four positive cases and four negative cases were identified. The maximum score was 25, the minimum score was 5, and the average score was 13. Two of the positive cases were located in the inguinal lymph nodes, one in the pleura, and one in the liver. In the group with local recurrences, four positive cases were identified, with a maximum score of 130, a minimum score of 5, and an average score of 63. All cases that tested positive for CD133 in both the metastases and local recurrence groups were pT4b at the initial diagnosis and were nodular melanomas (Table 4).

## 4. Discussion

CSCs represent a sub-population within tumors that possess the ability to self-renew and give rise to new tumor cells. Structurally, these cells share many characteristics with normal stem cells. This sub-population is believed to be crucial in tumor initiation, therapy resistance, and metastasis processes. The origin of these cells remains controversial, and no definitive mechanism has been identified in regard to their development [18]. One of the supported theories relates to that of mutations occurring at the level of normal stem cells, thus becoming tumor cells, but still possessing the properties of a stem cell [19]. Another hypothesis is that of cell regression, whereby tumor cells at the time of division undergo regression, and at least one of the daughter cells becomes tumor stem cells [20,21].

Identifying these tumor cells can be achieved through various methods, with immunohistochemistry being one of the most accessible techniques. This approach detects specific surface markers of cancer stem cells using targeted antibodies. Other identification methods include flow cytometry (based on physical properties), enzymatic activity analysis (e.g., ALDH), molecular testing (DNA and RNA sequencing), and functional analysis in vivo or in vitro [22,23].

CD133 (Prominin-1) is a transmembrane glycoprotein found on the surface of progenitor cells. It also serves as a marker for cancer stem cells, having been studied across various neoplastic lesions, including cutaneous melanoma [24]. The pathogenic activity of these cells in melanoma can be explained through multiple mechanisms: signaling pathways (Wnt, Notch, and Hedgehog) that promote survival and resistance to apoptosis, interaction with the tumor microenvironment to induce angiogenesis under hypoxic conditions, and cellular plasticity, which enables adaptation to different conditions [25].

Recent studies have demonstrated a correlation between CD133 expression on tumor cells and increased tumor aggressiveness, as reflected in poorer histological parameters, worsened prognosis, and higher recurrence or metastasis rates [26,27]. These associations were also observed in our study, highlighting the aggressiveness of CD133-positive cases. Some studies have shown increased CD133 expression in metastases and local recurrences, suggesting its role in tissue invasion and distant metastasis [28].

NANOG is an important transcription factor for maintaining stem cell status in the body. Numerous studies have demonstrated the importance of this factor in keeping the stem cell at an undifferentiated stage and regulating its activity in terms of renewal [29]. From an oncological point of view, the expression of the factor on a tumor cell has been associated with the identification of tumor stem cells. Studies in the literature have shown multiple roles of this transcription factor in terms of tumor cell activity, such as self-renewal that contributes to tumor heterogeneity, pluripotency, resistance to apoptosis, and the process of metastasis [30]. In our study, similar to the identified studies, we observed an expression of some cells with this marker, without having a statistically significant correlation in our case.

To quantify the immunohistochemical reactions for the two studied markers (CD133 and NANOG), the H-Score was used. This score was selected because it provides a semi-quantitative evaluation of expression, which is more accurate than a binary classification (positive/negative). It is also widely used in pathology, with clinical applications (e.g., hormone expression evaluation in breast cancer), and as a prognostic factor for other markers [31,32,33]. In our study, to avoid quantification and inter-observer errors, all immunohistochemical reactions were validated and verified by two pathologists.

The first analysis performed was for the NANOG marker, which statistically did not show any significant correlation. However, parameters such as the mean Breslow index, Pagetoid migration, and the presence of in situ lesions were identified, which showed increases compared to the reference group (H-Score 0), both within the H-Score Low group and the H-Score High group. These increases in tumor prognostic parameters could be suggestive of increased tumor aggressiveness and an increased ability to invade surrounding tissues. Regarding the expression analysis for CD133 in primary melanoma cases, in addition to the statistically significant correlations identified, a slight increase in the age of diagnosis and a slight increase in the average Clark level can be observed. Statistically significant changes (average Breslow index and the average mitotic count per 10/HPF) can be associated with a much more pronounced growth of tumors but also an increased aggressiveness.

We also analysed the correspondence between the positive expressions of these markers. Regarding the cases of primary cutaneous melanoma and local recurrences, no case was identified that showed positivity for both markers, an aspect that suggests the heterogeneity and differentiation of these cells. Among the cases of distant metastasis, two cases showed positivity for both markers, the first being a metastasis at the level of inguinal lymph nodes and the second being a metastasis at the pleural level. Both cases presented the pT4b stage at diagnosis.

The lack of statistical correlations between the H score, both for CD133 and NANOG, with some morphological parameters could suggest that the impact of these two proteins is not directly associated with melanoma progression, but at the same time underlines the plasticity and heterogeneity of these cells, properties by which they can adapt to various conditions and act in multiple ways.

Even though these proteins provide additional information about the behaviors and activity of tumor stem cells, their use for routine diagnostic purposes remains limited. Currently, these determinations are not specific enough to be used to guide routine diagnoses but may be used in the future to identify specific therapeutic targets.

Another very important role of these cells is their appearance in the early stages of the disease. By employing modern diagnostic techniques, these cells could be identified at a much earlier stage, allowing for a faster diagnosis of the disease. As a result, patients could benefit from more promptly administered therapy, as well as targeted treatment in cases where specific molecules relevant to the therapy are identified.

The main limitation of this study is the relatively small number of cases in which immunohistochemical reactions with anti-CD133 and anti-NANOG antibodies were performed. Moreover, a possible limitation of this study could be the lack of a long-term follow-up of patients or a cancer registry, which would provide a correlation between the expression of these markers and the evolution of patients post-therapy. Another limitation of this study is represented by the non-inclusion of cases of non-dysplastic nevi, a group of cases that could bring additional value to this study. However, this topic remains a future research direction for us.

Future directions for the development of this study include the expansion of patient groups, the analysis of other proteins present on the surface of these cells, and the inclusion of case groups with non-dysplastic nevi. It is also desired to achieve the long-term follow-up of patients, and the correlation of the results obtained from therapies received by the patients.

## 5. Conclusions

In conclusion, cutaneous melanoma case management, diagnosis, monitoring, and therapy are changing and improving day by day in accordance with new discoveries.

The results of our study regarding the expression of CD133 and NANOG markers in melanoma, both in cases of primary cutaneous melanoma and at the level of local recurrences and metastases, provide valuable information on the importance of these molecules in the activity of tumor stem cells. Having a statistically significant positivity for only one of the two markers highlights the variability, heterogeneity, and activity that characterize them. Our findings provide new insights into these tumor stem cells and may contribute to future therapies targeting these surface molecules.

## Figures and Tables

**Table 1:** H-Score for immunohistochemical reaction with anti-NANOG antibodies in patients with primary cutaneous melanoma.

	H-Score 0 n = 20	H-Score Low n = 8	H-Score Hig hn = 21
Average Age	60	63.5	67.66
Average Breslow	6.13	5.46	6.69
Ulceration			
YES	12 (60%)	2 (25%)	10 (47.62%)
NO	8 (40%)	6 (75%)	11 (52.38%)
Average Clark Level	4.3	4	3.39
Mitotic Count 10/HPF	25	25.2	17.8
Pagetoid Migration			
YES	16 (80%)	7 (87.5%)	18 (85.71%)
NO	4 (20%)	1 (12.5%)	3 (14.29%)
In Situ Lesion			
YES	8 (40%)	8 (100%)	16 (76.19%)
NO	12 (60%)	0 (0%)	5 (23.81%)
Average Ki-67	50%	40%	42%

**Table 2:** H-Score for immunohistochemical reaction with anti-NANOG antibodies in patients with metastases and local recurrences.

	Metastases n = 10	Local Recurrences n = 6
Positive Cases	8 (80%)	4 (66%)
Average H-Score	43	31
Min Score	5	5
Max Score	125	60
Negative Cases	2 (20%)	2 (33%)

**Table 3:** H-Score for immunohistochemical reaction with anti-CD133 antibodies in patients with primary cutaneous melanoma.

	H-Score = 0 n = 44	H-Score > 0 n = 6
Average Age	63.81	65.83
Average Breslow	5.82	10.45
Ulceration		
YES	20 (45.45%)	4 (66%)
NO	22 (54.55%)	2 (33%)
Average Clark Level	4.09	4.33
Mitotic Count 10/HPF	19.35	35.5
Pagetoid Migration		
YES	38 (86.36%)	4 (66%)
NO	6 (13.64%)	2 (33%)
In Situ Lesion		
YES	28 (63.63%)	4 (66%)
NO	16 (36.37%)	2 (33%)
Average Ki-67	43.8%	48.3%

**Table 4:** H-Score for immunohistochemical reaction with anti-CD133 antibodies in patients with metastases and local recurrences.

	Metastases	Local Recidives
Positive Cases	4 (40%)	4 (66%)
Average H-Score	13	63
Min Score	5	5
Max Score	25	130
Negative Cases	6 (60%)	2 (33%)

## Data Availability

All data are available and can be produced upon request.
